# Supplementary material for: Recent co-evolution of two pandemic plant diseases in a multi-hybrid swarm
Source: Nat Ecol Evol. 2023 Nov 9;7(12):2055–66. doi: 10.1038/s41559-023-02237-z (PMC10697843; doi:10.1038/s41559-023-02237-z)
Supplement: Supplementary file 1 — Reporting Summary [file 41559_2023_2237_MOESM1_ESM.pdf]

nature portfolio

Corresponding author(s): Mark L. Farman

Last updated by author(s): Apr 20, 2023

Reporting Summary

Nature Portfolio wishes to improve the reproducibility of the work that we publish. This form provides structure for consistency and transparency in reporting. For further information on Nature Portfolio policies, see our Editorial Policies and the Editorial Policy Checklist.

Statistics

For all statistical analyses, confirm that the following items are present in the figure legend, table legend, main text, or Methods section.

n/a	Confirmed	
☒	☐	The exact sample size (*n*) for each experimental group/condition, given as a discrete number and unit of measurement
☒	☐	A statement on whether measurements were taken from distinct samples or whether the same sample was measured repeatedly
☒	☐	The statistical test(s) used AND whether they are one- or two-sided<br>*Only common tests should be described solely by name; describe more complex techniques in the Methods section.*
☒	☐	A description of all covariates tested
☒	☐	A description of any assumptions or corrections, such as tests of normality and adjustment for multiple comparisons
☒	☐	A full description of the statistical parameters including central tendency (e.g. means) or other basic estimates (e.g. regression coefficient) AND variation (e.g. standard deviation) or associated estimates of uncertainty (e.g. confidence intervals)
☒	☐	For null hypothesis testing, the test statistic (e.g. *F*, *t*, *r*) with confidence intervals, effect sizes, degrees of freedom and *P* value noted<br>*Give P values as exact values whenever suitable.*
☐	☒	For Bayesian analysis, information on the choice of priors and Markov chain Monte Carlo settings
☒	☐	For hierarchical and complex designs, identification of the appropriate level for tests and full reporting of outcomes
☒	☐	Estimates of effect sizes (e.g. Cohen's *d*, Pearson's *r*), indicating how they were calculated

Our web collection on statistics for biologists contains articles on many of the points above.

Software and code

Policy information about availability of computer code

Data collection	no software was used for data collection
Data analysis	Trimmomatic 0.39, velvet 1.2.10, Newbler 2.9/3.0, Bowtie2 2.2.5, Genome Analysis Tool Kit 4.1.3.0, picard 2.21.6, ChromoPainterV2,MEGA X, RAxML-NG 0.9.0, Partition finder 2.1.1, BEAST 2.9, ShinyHaplotypes 2, and various custom code (available on GitHub)

For manuscripts utilizing custom algorithms or software that are central to the research but not yet described in published literature, software must be made available to editors and reviewers. We strongly encourage code deposition in a community repository (e.g. GitHub). See the Nature Portfolio guidelines for submitting code & software for further information.

Data

Policy information about availability of data

All manuscripts must include a data availability statement. This statement should provide the following information, where applicable:

- Accession codes, unique identifiers, or web links for publicly available datasets
- A description of any restrictions on data availability
- For clinical datasets or third party data, please ensure that the statement adheres to our policy

Data availability
Sequence data are available under various NCBI accessions as listed in
Supplementary Data File 1. Some of the datasets used for the analyses described herein are available at https://github.com/drdna/WheatBlastEvolution. Datasets

that are too large to be uploaded to public repositories can be provided upon request from the communicating author.
Code availability
Custom bash, perl and R codes used to perform the analyses and generate figures are available on GitHub: (https://github.com/drdna/WheatBlastEvolution).

Research involving human participants, their data, or biological material

Policy information about studies with human participants or human data. See also policy information about sex, gender (identity/presentation), and sexual orientation and race, ethnicity and racism.

Reporting on sex and gender	*Use the terms sex (biological attribute) and gender (shaped by social and cultural circumstances) carefully in order to avoid confusing both terms. Indicate if findings apply to only one sex or gender; describe whether sex and gender were considered in study design; whether sex and/or gender was determined based on self-reporting or assigned and methods used. Provide in the source data disaggregated sex and gender data, where this information has been collected, and if consent has been obtained for sharing of individual-level data; provide overall numbers in this Reporting Summary. Please state if this information has not been collected. Report sex- and gender-based analyses where performed, justify reasons for lack of sex- and gender-based analysis.*
Reporting on race, ethnicity, or other socially relevant groupings	*Please specify the socially constructed or socially relevant categorization variable(s) used in your manuscript and explain why they were used. Please note that such variables should not be used as proxies for other socially constructed/relevant variables (for example, race or ethnicity should not be used as a proxy for socioeconomic status). Provide clear definitions of the relevant terms used, how they were provided (by the participants/respondents, the researchers, or third parties), and the method(s) used to classify people into the different categories (e.g. self-report, census or administrative data, social media data, etc.) Please provide details about how you controlled for confounding variables in your analyses.*
Population characteristics	*Describe the covariate-relevant population characteristics of the human research participants (e.g. age, genotypic information, past and current diagnosis and treatment categories). If you filled out the behavioural & social sciences study design questions and have nothing to add here, write "See above."*
Recruitment	*Describe how participants were recruited. Outline any potential self-selection bias or other biases that may be present and how these are likely to impact results.*
Ethics oversight	*Identify the organization(s) that approved the study protocol.*

Note that full information on the approval of the study protocol must also be provided in the manuscript.

Field-specific reporting

Please select the one below that is the best fit for your research. If you are not sure, read the appropriate sections before making your selection.

☐ Life sciences ☐ Behavioural & social sciences ☒ Ecological, evolutionary & environmental sciences

For a reference copy of the document with all sections, see nature.com/documents/nr-reporting-summary-flat.pdf

Ecological, evolutionary & environmental sciences study design

All studies must disclose on these points even when the disclosure is negative.

Study description	The experiment was designed so as to determine the genetic basis for accumulation of enormous quantities of population variation over an extremely short period of time. We used 90 fungal isolates from the study population and 96 representing other host-specialized forms. We initially used genome alignments to characterize the variation and then various population genomic tools to identify, characterize and quantity genetic admixture events. Finally, we used Bayesian methods to estimate the time scale for the new population's evolution.
Research sample	Contemporarily-sampled fungal strains collected from regions exhibited outbreaks of disease caused by Pyricularia species; historical samples from culture collections; genomic data from the global research community - either downloaded from national data repositories, or provided by individuals scientists.
Sampling strategy	Contemporary samples and historical were collected from regions exhibiting wheat blast outbreaks; data were gathered from all available sources, with the goal of maximizing representation of Pyricularia isolates from as many host genera/species as possible
Data collection	Data were collected automatically by sequencing machines, or downloaded from online repositories. Associated metadata were gathered at the same time.
Timing and spatial scale	Data were gathered continuously from 2008 to 2020
Data exclusions	the only data that were excluded were poor quality genome assemblies that yielded an unacceptable high number of false genotyping calls

Reproducibility	Each result is supported by multiple, independent lines of evidence. All data, no matter what tools/analytical approaches were fully consistent
Randomization	N/A
Blinding	N/A

Did the study involve field work? ☐ Yes ☒ No

Reporting for specific materials, systems and methods

We require information from authors about some types of materials, experimental systems and methods used in many studies. Here, indicate whether each material, system or method listed is relevant to your study. If you are not sure if a list item applies to your research, read the appropriate section before selecting a response.

Materials & experimental systems

n/a	Involved in the study
☒	☐ Antibodies
☒	☐ Eukaryotic cell lines
☒	☐ Palaeontology and archaeology
☒	☐ Animals and other organisms
☒	☐ Clinical data
☒	☐ Dual use research of concern
☒	☐ Plants

Methods

n/a	Involved in the study
☒	☐ ChIP-seq
☒	☐ Flow cytometry
☒	☐ MRI-based neuroimaging
